# Love Wave Sensors with Silver Modified Polypyrrole Nanoparticles for VOCs Monitoring

**DOI:** 10.3390/s20051432

**Published:** 2020-03-06

**Authors:** Milena Šetka, Fabio A. Bahos, Daniel Matatagui, Isabel Gràcia, Eduard Figueras, Jana Drbohlavová, Stella Vallejos

**Affiliations:** 1 CEITEC—Central European Institute of Technology, Brno University of Technology, 61200 Brno, Czech Republic; milena.setka@ceitec.vutbr.cz (M.Š.); jana.drbohlavova@ceitec.vutbr.cz (J.D.); 2 Instituto de Ciencias Aplicadas y Tecnología (ICAT), Universidad Nacional Autónoma de México, Ciudad Universitaria, Ciudad de México 04510, Mexico; fbahos@unicauca.edu.co (F.A.B.); daniel.matatagui@icat.unam.mx (D.M.); 3 SENSAVAN, Instituto de Tecnologías Físicas y de la Información (ITEFI, CSIC), Serrano 144, 28006 Madrid, Spain; 4 Instituto de Microelectrónica de Barcelona (IMB-CNM, CSIC), Campus UAB, 08193 Bellaterra, Spain; isabel.gracia@imb-cnm.csic.es (I.G.); eduard.figueras@imb-cnm.csic.es (E.F.)

**Keywords:** polypyrrole, gas sensors, love wave sensors, volatile organic compounds

## Abstract

Love wave sensors with silver-modified polypyrrole nanoparticles are developed in this work. These systems prove functional at room temperature with enhanced response, sensitivity and response time, as compared to other state-of-the-art surface acoustic wave (SAW) sensors, towards volatile organic compounds (VOCs). Results demonstrate the monitoring of hundreds of ppb of compounds such as acetone, ethanol and toluene with low estimated limits of detection (~3 ppb for acetone). These results are attributed to the use of silver-modified polypyrrole as a second guiding/sensitive layer in the Love wave sensor structure, which provides further chemically active sites for the gas-solid interactions. The sensing of low VOCs concentrations by micro sensing elements as those presented here could be beneficial in future systems for air quality control, food quality control or disease diagnosis via exhaled breath as the limits of detection obtained are within those required in these applications.

## 1. Introduction

Volatile organic compounds (VOCs) are low-molecular-weight organic compounds and most of them are categorized as harmful substances which may have a short term and/or long term negative impact on the environment (e.g., creation of tropospheric ozone) and on health (e.g., allergic reactions, asthma, damage of central nervous system, cancer, etc.) [1]. Hence, currently, there is an increased need of VOCs monitoring for outdoor and indoor air quality control (e.g., VOCs emission from industrial processes, traffic activity, waste treatment and disposal, tobacco smoking, etc.), food quality analysis (e.g., VOCs linked to food spoilage) and health diagnosis (e.g., VOCs associated to disease biomarkers) [1,2,3,4,5]. Therefore, the sensitive and selective detection of low VOCs concentrations using simple and low-cost sensors may have a significant effect on quality of life.

Surface acoustic wave (SAW) sensors have attracted great attention thanks to their sensitivity, low limits of detection (LOD), room temperature operation, low power consumption, relatively simple architecture, small dimensions and ability to work in wireless mode [6]. Moreover, previous literature on SAW sensors states the feasibility to sense gases and vapors by using sensitive materials such as semiconducting metal oxides [7], polymers [8], graphene oxide [9] and carbon nanotubes [10]. Apart from the competing advantages of those sensing materials, polymers-based SAW structures demonstrated improved performance in gas sensing, owing to their low density and shear velocity. Additionally, polymers also have high sensitivity at room temperature (as opposed to semiconducting metal oxides) and report good cost-effectiveness (compared to carbon based materials) [6,11]. Among various types of SAW structures (e.g., Rayleigh, shear horizontal SAW, Love, leaky), those based on Love waves (L-SAW) have been identified to have high mass sensitivity, generally, due to the surface confinement of energy in the thin guiding layer, which makes the surface extremely sensitive to any perturbations [12].

Polypyrrole (PPy) and its sensing properties to VOCs have been investigated in the past typically by implementing this conductive polymer into chemo-resistive structures [13]. In contrast, the use of PPy in the field of SAW sensors and especially in L-SAW sensors has been rarely described in the literature, in which is found a significantly low amount of reports for L-SAW based PPy structures. Our recent studies, however, indicate that L-SAW sensors with multi-guiding layers containing gold modified PPy as a guiding and gas sensitive material can enhance to a higher degree the sensing performance at room temperature of this sensor as compared to other SAW or chemo-resistive structures with similar PPy modification [14].

In this context, this work explores further the use of L-SAW structures with second guiding/sensitive layers based on silver-modified polypyrrole (Ag/PPy). The work also evaluates the performance of these structures to various VOCs (including ethanol, acetone and toluene), as these vapors are of interest in multiple applications, such as human breath analyzers, food quality analyzers and/or environment monitoring equipment.

## 2. Materials and Methods

### 2.1. L-SAW Sensor

The fabrication process of the L-SAW platforms was adopted from our previous works [14,15]. Briefly, the two interdigital transducers (IDTs) ports (input and output) were patterned on the surface of a piezoelectric substrate (ST-90°X quartz). Each port of the IDTs consisted of 75 pairs of double-electrode type aluminium electrodes with a periodicity of 28 μm. The delay line and IDTs aperture were set to 2.1 mm. In this work, a multi-guiding layer structure, in which the first guiding layer consisted of SiO_2_ (3 μm thick) and the second guiding layer of a gas sensitive conductive polymer, was employed. The second guiding and gas sensitive layer was formed from either PPy or Ag/PPy nanoparticles (NPs). To integrate the gas sensitive material over the L-SAW platform, both PPy and Ag/PPy were spin coated at a velocity of 2500 rpm. To control the reproducibility of the sensing layer, the L-SAW substrates were placed into a customized holder built to keep the substrates in a fixed and aligned position during the coating process.

**Figure 1:**
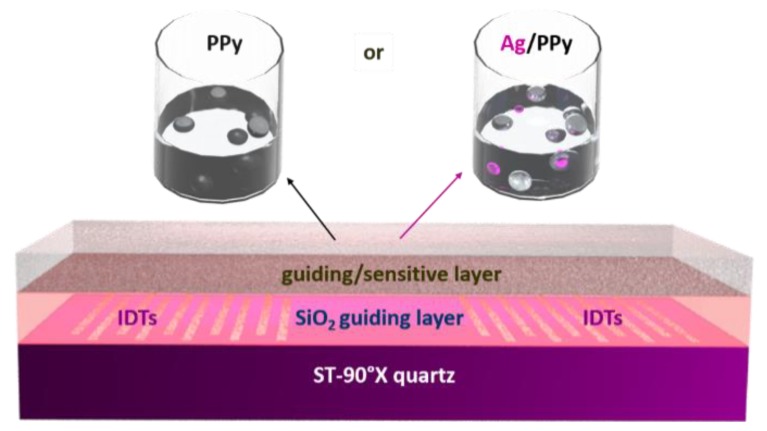
Schematic view of a L-SAW sensor and solutions used for deposition of PPy or Ag/PPy guiding/sensitive layers.

PPy NPs were synthesized by an oxidative chemical polymerization as described in our previous work [14], whereas Ag NPs were synthesized by a chemical reduction of AgNO_3_ with NaBH_4_ using the conditions reported previously [16]. The Ag/PPy NPs solution consisted of a mixture of Ag NPs and PPy NPs in a volumetric ratio of 1:10 since we noticed previously that this relation of catalytic metal and polymer is adequate for both the uniform coating and the sensing properties [14]. The morphology and chemical composition of PPy and Ag NPs were investigated using a high-resolution transmission electron microscope (HR-TEM, FEI TITAN Themis 60–300 kV), Scanning Electron Microscope equipped with focus ion beam (FIB/SEM, Helios G4 NanoLab DualBeam™) and X-ray photoelectron spectroscopy (XPS, Kratos Axis Supra). The electrical characterization and measurements of the transmission scattering parameter (S_21_) were performed using a network analyser (Agilent 4395A). Figure 1 shows a schematic view of an L-SAW sensor and the two gas sensitive materials deposited by spin coating.

### 2.2. Gas Sensing Test

Gas sensing tests of the L-SAW sensors (based on PPy and Ag/PPy) were carried out simultaneously in a continuous flow system equipped with mass flow controllers and calibrated gas cylinders of acetone (Praxair, 100 ppm), ethanol (Praxair, 100 ppm) and toluene (Praxair, 200 ppm), as described in our previous work [17]. The sensors were tested towards these gases in a concentration range from 0.5 to 5 ppm, both in dry and humid environments with 10% and 30% relative humidity (RH). The sensors were exposed to each analyte for 2 min and purified with dry or humid synthetic air for 10 min at a constant temperature of 24 °C. The moisture and temperature inside the gas chamber were monitored using a humidity/temperature sensor (SHT71, operating from 0 to 100% RH, accuracy of ±3% RH). The sensing test was performed over a 10 days period with a continuous operation of 10 h per day. The sensor response was measured using an electronic measurement system equipped with an amplifier, directional coupler and frequency counter [18]. The response (frequency shift) was defined as the difference in the resonant frequency produced by the exposure of the sensors to dry/humid air and the target analyte. The response time was defined as the time required to reach 90% of the total frequency shift value, whereas the recovery time was defined as the time required to return to 90% of the baseline frequency after the target gas was purged.

## 3. Results

### 3.1. Gas Sensitive Material Properties

**Figure 2:**
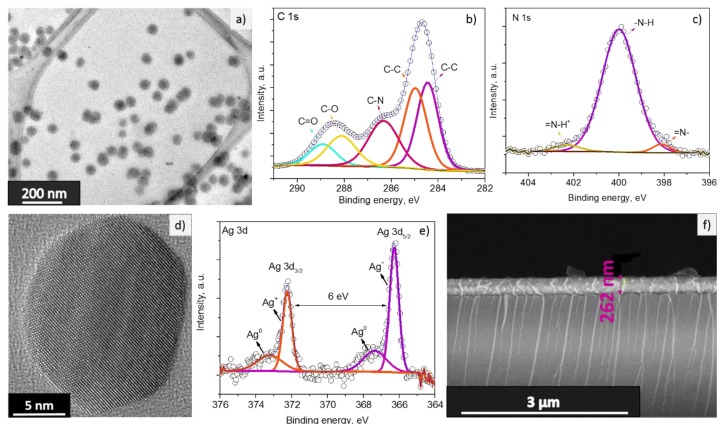
TEM image of PPy NPs (**a**); high-resolution XPS spectra of the C 1s (**b**) and N 1s (**c**) core levels at the PPy NPs; HR-TEM image of the Ag NPs (**d**); high resolution XPS spectra of the Ag 3d core levels at the Ag NPs (**e**); typical cross-section SEM image of the L-SAW sensors after spin coating of PPy or Ag/PPy NPs (**f**).

The morphology and chemical composition of the gas sensitive materials (i.e., PPy and Ag/PPy) were examined by TEM and XPS. TEM of the PPy NPs (Figure 2a) revealed spherical shaped particles with an average size of 44 ± 10 nm, calculated for a population of 100 particles. XPS displayed characteristic C 1s (Figure 2b) and N 1s (Figure 2c) core level spectra. The components in the C 1s spectrum centred at 284.4, 285.0, 286.3, 288.1 and 288.9 eV are ascribed to the binding energies of the C–C bonds of β atoms, the C–C bonds of α atoms and the C–N, C–O and C=O bonds of PPy, respectively. Similarly, the three components in the N 1 s spectrum correspond to the =N– (398.0 eV), –N–H (400.0 eV) and =N–H^+^ (402.3 eV) bonds of PPy. The presence of these components in the C 1s and N 1s core level spectra is consistent with our previous observations [19] and corroborates the synthesis of PPy.

The synthesis of crystalline Ag NPs was also corroborated by HR-TEM. Figure 2d displays the TEM images of the particles with mean diameter of approximately 17 ± 3 nm (calculated for a population of 30 particles) and lattice fringes with spacing of ~0.23 nm, consistent with the (111) planes of Ag face centered cubic phase (JCPDS number 04-0783) [20]. High-resolution XPS analysis of the Ag NPs (Figure 2e) revealed typical Ag 3d doublets separated by 6 eV. The deconvoluted Ag 3d doublet with a pair of components centered at 366.3 and 372.3 eV, and another pair centered at 367.4 and 373.2 eV indicates the presence of Ag^+^ and Ag^0^, respectively. This suggests the coexistence of silver oxide (Ag_2_O) and metallic silver (Ag), in accordance with previous observations [21].

Further SEM study of the PPy and Ag/PPy NPs guiding/sensitive layers after spin coating showed the integration of uniform sensing layers with a thickness of ~262 ± 10 nm on the L-SAW platforms. A typical cross section SEM image of the L-SAW sensors is displayed in Figure 2f.

### 3.2. Electrical Characterization of the L-SAW Sensors

**Figure 3:**
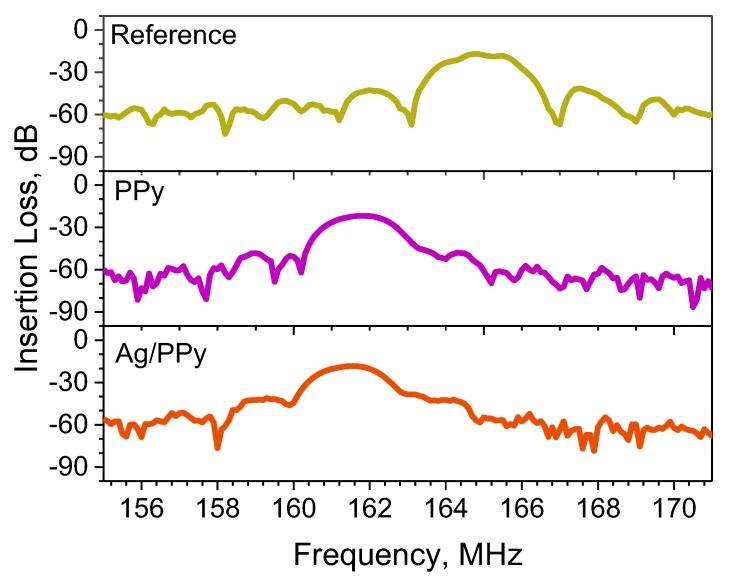
Transmission scattering parameters (S_21_) of the uncoated (reference) and coated (with PPy or Ag/PPy sensitive layer) L-SAW structures.

The PPy and Ag/PPy L-SAW sensors were electrically characterized by measuring the transmission scattering parameter (S_21_). These results were compared to a reference L-SAW platform with only a SiO_2_ guiding layer to determine the frequency shifts after the incorporation of the second guiding/sensitive layer. Figure 3 illustrates the resonant frequency and insertion loss of the reference, PPy and Ag/PPy L-SAW sensors. These results show that the operating frequency and insertion loss of the reference L-SAW sensor are 165.1 MHz and −16 dB, respectively. The figure also shows the shift in the resonance frequency of the PPy and Ag/PPy sensors to lower numbers (161.6 ± 0.1 MHz) with respect to the reference, as well as the increase of the insertion loss (19.7 ± 1 dB). Both these changes are consistent with the use of the PPy and Ag/PPy layers as a second guiding layer. The relatively low insertion losses in the double guiding layer L-SAW structures lead to sensors with low overall noise (lower than 10 Hz) and, in turn, low LOD.

### 3.3. Gas Sensing Properties of the L-SAW Sensors

**Figure 4:**
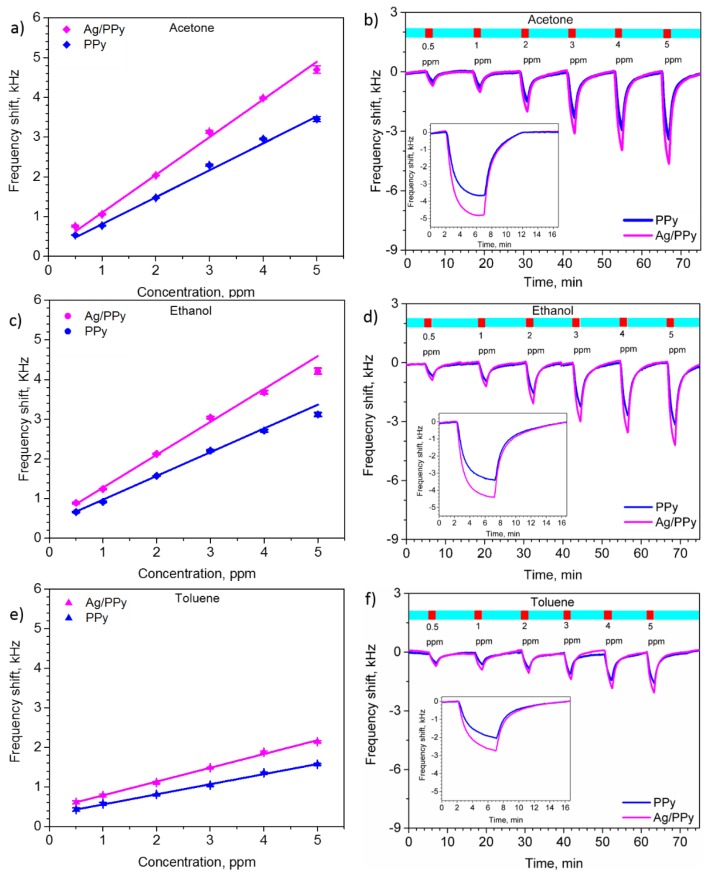
Calibrations curves and dynamic responses of the L-SAW sensors (based on PPy and Ag/PPy) for acetone (**a**,**b**), ethanol (**c**,**d**), and toluene (**e**,**f**). The insets in (**b**,**d**,**f**) display the time dependent response to 5 ppm of each gas.

The sensing capability of the L-SAW sensors (PPy and Ag/PPy) was evaluated by exposing these devices simultaneously to various concentrations (between 0.5 and 5 ppm) of acetone, ethanol and toluene at room temperature. The calibration curves for acetone, ethanol and toluene are shown in Figure 4a,c,e, respectively. These figures show the proportional increase of the sensor response with the increase of concentration for each gas without reaching the saturation point, which indicates the possibility to sense higher concentrations of these analytes. Generally, the results display enhanced responses for the Ag/PPy sensors compared to the PPy sensors for all tested gases, with frequency shifts of approximately 1.4 times more for the Ag/PPy sensors than for the PPy sensors. These results are in line with previous experimental research, which proved the enhancement of gas sensing properties (e.g., sensors response) by the modifications of a host gas sensitive material with metal catalysts (e.g., Ag, Au) due to a “spillover effect” [14,22].

The LOD of the Ag/PPy L-SAW sensors (defined as the concentration providing a signal-to-noise ratio of at least three [23]) was estimated to be 3, 5 and 20 ppb for acetone, ethanol and toluene, respectively. These LOD are below the limits set for acetone, ethanol and toluene in different areas. For instance, the American Conference of Governmental Industrial Hygienists (ACGIH) sets the occupational threshold limit values (TLV) for acetone, ethanol and toluene to 250, 1000 and 20 ppm, respectively, considering 8-h time-weighted averages. Similarly, the concentration of VOCs in the food industry are above (typically by tens or hundreds of ppb) the LOD obtained for our sensors [24]. Additionally, in the breath analysis field, the concentrations of acetone, ethanol and toluene in the exhaled breath (e.g., lung cancer patients register concentrations of 112–2654 ppb of acetone, 13–1520 ppb of ethanol and 9.3–21.3 ppb of toluene) [4] are above the LOD obtained in this work.

The dynamic response of the PPy and Ag/PPy L-SAW sensors exposed to various concentrations of acetone, ethanol and toluene are presented in Figure 4b,d,f, respectively. One can notice from these results that the Ag/PPy and PPy sensors displayed stable and reversible responses. In order to reach the steady state of the response and determine the response and recovery time, the sensors were exposed to a gas concentration of 5 ppm for 5 min and then the gas was purged with dry synthetic air for 10 min (see the inset responses in Figure 4b,d,f). Overall, the response and recovery time for the Ag/PPy sensors was approximately 10 s faster than that for the PPy sensors. This could be attributed the catalytic properties of chemically active Ag NPs, which accelerate the gas-solid interactions at the guiding/sensitive film. These results also reveal faster response and recovery time to acetone than to ethanol or toluene for both the PPy and Ag/PPy sensors. The response time for the Ag/PPy sensors to acetone and ethanol, for instance, was below 2.5 min, while the response time to toluene was below 3.5 min. In addition, the Ag/PPy sensors required less than 4 min to recover after acetone exposure and less than 5.5 min after ethanol and toluene exposure.

Results in Figure 4b,d,f also give evidence of the negative frequency shift of the response to acetone, ethanol and toluene. This decrease in the resonant frequency of the L-SAW sensors may indicate that the mass and acoustoelectric loading effects outweigh the elastic effect [25]. The mass loading effect and therefore the decrease of the resonant frequency of the L-SAW sensors may be connected to the change in the mass of the PPy and Ag/PPy guiding/sensitive layer caused by the sorption of gas analytes (i.e., acetone, ethanol and toluene). The acoustoelectric effect, in contrast, may be connected to the adsorption of the gas analytes at the guiding/sensitive layer and the increase of the conductivity of PPy [26], which is also characterized by a decrease in the resonant frequency of the sensor. The contribution of the elastic effect, which is generally observed by an increase in the resonant frequency, is ruled out in this particular case [25].

**Figure 5:**
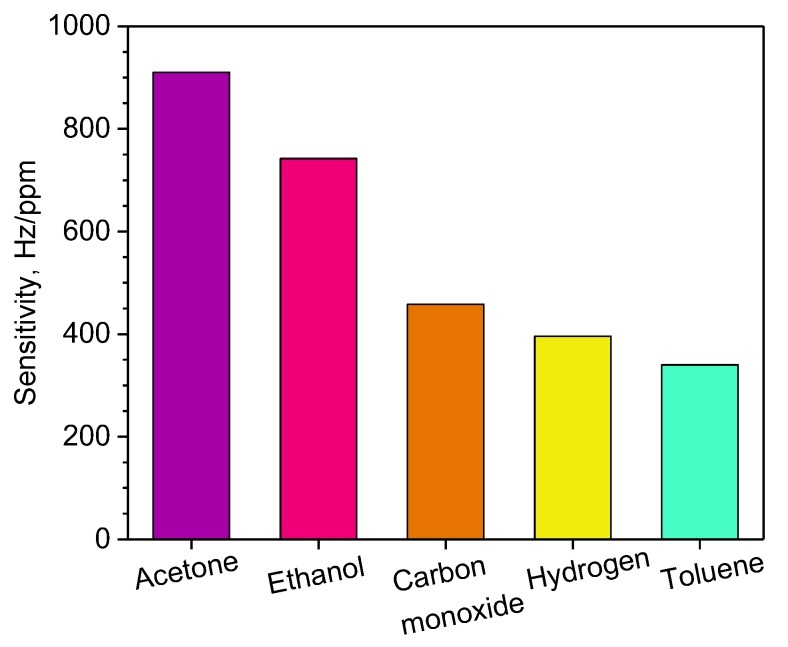
Sensitivity of Ag/PPy L-SAW sensors to acetone, ethanol, carbon monoxide, hydrogen and toluene for concentrations between 0.5 and 5 ppm.

As the Ag/PPy L-SAW sensors showed improved sensing properties over the PPy sensors, further analysis was performed on these sensors. Figure 5 shows the sensitivity of the Ag/PPy sensors calculated as the ratio between the change in frequency response of the sensor and the change in the gas concentration. These results show the sensitivity to various gases, including acetone (910 Hz/ppm), ethanol (742 Hz/ppm), carbon monoxide (458 Hz/ppm), hydrogen (396 Hz/ppm) and toluene (340 Hz/ppm). Overall, the sensors displayed good stability keeping a constant operating frequency and reproducible responses with standard errors below 5% after testing all gases in dry ambient. One can notice in Figure 5 that the Ag/PPy sensors exhibit a higher sensitivity to acetone than to other analytes such as ethanol and toluene, which register a cross-sensitivity respect to acetone of 81% and 37%, respectively. Similarly, the cross-sensitivity of other gases such as carbon monoxide and hydrogen with respect to acetone is found to be 50% and 43%, respectively. In summary, these results indicate a relatively low interference among the tested analytes.

The performance of the Ag/PPy L-SAW gas sensors investigated in this work were summarized and compared with other SAW gas sensors reported in the literature (Table 1). Results indicate that our sensors exhibited significantly higher sensitivity as well as lower LOD to acetone, ethanol and toluene in comparison to other SAW systems. The lowest tested concentrations of those VOCs detected via Ag/PPy L-SAW sensors were crucially lower than the theoretical LOD of state-of-the art sensors (Table 1). This demonstrates the capability of Ag/PPy based Love wave sensors to detect ppb levels of the target VOCs.

**Figure 6:**
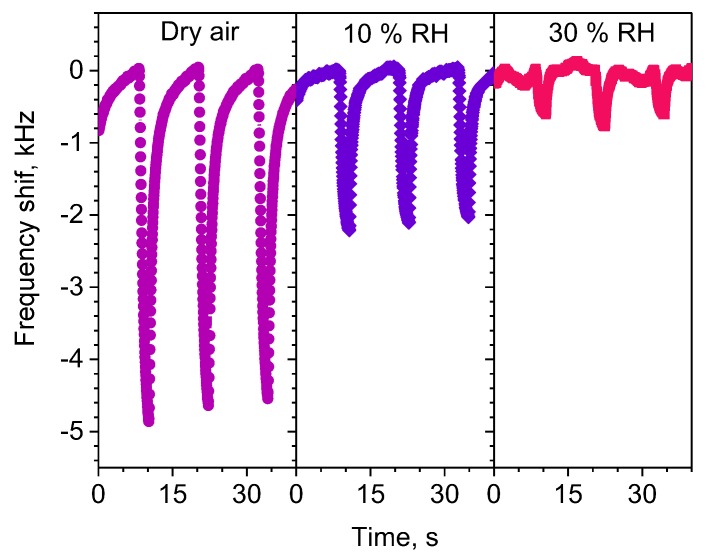
Frequency response of Ag/PPy L-SAW sensor to acetone concentration of 5 ppm under dry air, 10% and 30% relative humidity (RH).

To evaluate the effect of humidity on the sensing response, the Ag/PPy sensors were tested to acetone at RH of 10% and 30%. Figure 6 compares the frequency shift of the sensor to 5 ppm of acetone in dry and humid conditions with 10% and 30% RH. The tests show that the sensor response decreases by a factor of 2 and 7 when the atmosphere changes from dry to 10% and 30% RH, respectively. Humidity tests are generally not reported for state-of-the art SAW sensors; therefore, these characteristics have not been included in Table 1. The loss of response in the Ag/PPy sensors in a humid atmosphere may be caused by the water vapor sorption into the polymer layer, which fills the free volume fraction in the polymer and reduces the gas permeability [31]. Additionally, after the humidity test, we registered a decrease in the sensor response in dry ambient of about 35% compared to the responses obtained in the initial operation hours (notice that the same sensors were exposed to all target gases in a dry and humid environment accumulating an operation time of 100 h). The irreversible loss of response in the material may be caused by the humidity rather than the testing time, as during the tests in dry ambient the response registered low dispersion as described above. This is a common issue in polymer based gas sensors that needs further technological solutions such as the use of humidity filters or dehydration elements [32] in order to exploit these sensors in future consumer devices.

## 4. Conclusions

This work reports the properties of non-modified and silver-modified PPy L-SAW sensors at room temperature for detection of ppb-levels of VOCs, including acetone, ethanol and toluene. The above results suggest that PPy functionalization using Ag NPs enhances the response, sensitivity and speed of L-SAW sensors to organic vapors, particularly to acetone (910 Hz/ppm). A moderate response to acetone was also registered by running the tests in humid conditions (10% and 30% RH) with the sensors detecting concentrations down to 1 ppm. Overall, these results demonstrated enhanced properties compared to other state-of-the-art SAW sensors, providing a technological solution for monitoring low concentrations of VOCs at room temperature.

## Figures and Tables

**Table 1:** Comparative table of state-of the-art SAW sensors and Ag/PPy L-SAW sensors for acetone, ethanol and toluene.

Sensitive Material	Gas	LTC (ppm)	HTC (ppm)	Sensitivity (Hz/ppm)	LOD (ppm)	Temperature	Reference
Ag/PPy	Acetone	0.5	5	910	0.003	RT	This work
	Ethanol			742	0.005		
	Toluene			340	0.020		
PPy	Acetone	5.5	80	116.4	-	RT	[27]
PEI/Fe_3_O_4_	Ethanol	160	16,000	1.6	65	ND	[28]
	Toluene			1.9	54		
MWCNTs-PEI	Ethanol	200	40,000	1.2	176.5	ND	[29]
	Toluene			1.2	170.7		
PEI/WO_3_	Acetone	50	800	3.4	15	RT	[8]
	Ethanol			7.9	6		
	Toluene			4.8	11		
GO	Ethanol	30	750	30	-	ND	[9]
	Toluene			24	-		
ZIF/Au	Acetone	5	25	28	1.1	RT	[30]
	Ethanol			72	0.5		
SnO_2_/Co_3_O_4_	Toluene	100	900	0.6	50	RT	[7]
PEUT-MWCNTs	Toluene	25	200	12.2	0.6	RT	[10]

LTC—lowest tested concentration, HTC—highest tested concentration, RT—room temperature, MWCNTs—multi-wall carbon nanotubes, PEI—polyethylenimine, GO—graphene oxide, ZIF—zeolitic imidazolate frameworks, PEUT—polyetherurethane, ND—not defined.
